# The Application of Stem Cells and Exosomes in Promoting Nerve Conduits for Peripheral Nerve Repair

**DOI:** 10.34133/bmr.0160

**Published:** 2025-04-14

**Authors:** Mengen Li, Ye Tang, Chengkai Zhou, Yan Geng, Chenxi Zhang, Yuwei Hsu, Le Ma, Wei Guo, Ming Li, Yanhua Wang

**Affiliations:** ^1^ National Center for Trauma Medicine, Beijing 100044, China.; ^2^ Key Laboratory of Trauma and Neural Regeneration, Ministry of Education, Peking University, Beijing 100044, China.; ^3^ Trauma Medicine Center, Peking University People’s Hospital, Beijing 100044, China.; ^4^ Department of Orthopedics and Trauma, Peking University People’s Hospital, Beijing 100044, China.; ^5^ Emergency Department, Peking University People’s Hospital, Beijing 100044, China.

## Abstract

The repair of peripheral nerve injury (PNI) presents a multifaceted and protracted challenge, with current therapeutic approaches failing to achieve optimal repair outcomes, thereby not satisfying the considerable clinical demand. The advent of tissue engineering has led to a growing body of experimental evidence indicating that the synergistic application of nerve conduits, which provide structural guidance, alongside the biological signals derived from exosomes and stem cells, yields superior therapeutic results for PNI compared to isolated interventions. This combined approach holds great promise for clinical application. In this review, we present the latest advancements in the treatment of PNI through the integration of stem cells or exosomes with nerve conduits. We have addressed the inadequate efficiency of exosomes or stem cells in conjunction with nerve conduits from 3 perspectives: enhancing stem cells or exosomes, improving nerve conduits, and incorporating physical stimulation.

## Introduction

PNI represents a prevalent clinical neurological condition that impacts millions of individuals globally each year [1]. The current understanding of the regenerative process following PNI is delineated into 3 primary stages. Initially, the axons at the site of injury disconnect from the neuronal cell body, leading to the gradual degeneration of distal axons through a process known as Wallerian degeneration [2]. In the subsequent stage, Schwann cells (SCs) undergo dedifferentiation into progenitor-like cells, which facilitate the recruitment of macrophages and other cellular entities to clear debris and modify the microenvironment [3]. Ultimately, these cells collaborate to form a tubular structure referred to as a nerve bridge, which serves to guide axonal regeneration [4].

To facilitate the repair of peripheral nerves, clinical interventions are categorized into nonsurgical and surgical approaches. Nonsurgical treatments encompass the application of physical stimuli (including electrical, magnetic, laser, and ultrasound modalities), as well as the use of growth factors, cellular sources, and various proteins, primarily serving as adjunctive therapies to surgical interventions, particularly for smaller PNI [5]. Surgical grafting is the predominant treatment modality for extensive nerve injuries, with graft options including autologous nerves, allogeneic nerves, decellularized nerve grafts, and bioengineered synthetic conduits. Presently, autologous nerve grafting is regarded as the gold standard for surgical repair [6]. However, it presents several challenges in clinical practice, such as limited availability of donor tissues, increased surgical trauma, and a heightened risk of neuroma formation [7]. Consequently, researchers are actively investigating alternatives to autologous nerve grafting.

Among these options, bioartificial synthetic conduits have several benefits, including as customizability, simplicity of insertion, and simplified manufacturing and sterilizing procedures [8–10]. Therefore, the application of bioartificial synthetic conduits in peripheral nerve repair, specifically as nerve conduits, holds great promise and potential. Nonetheless, the clinical efficacy of nerve conduits remains inferior to that of autologous nerve grafts, primarily due to limitations in bioactivity and challenges related to immune compatibility [11]. Research has indicated that stem cells possess paracrine and differentiation capabilities, demonstrating beneficial effects in tissue repair [12]. Exosomes, which are the principal secretory products through which cells exert paracrine effects, can transport bioactive molecules and exhibit biocompatibility with low immunogenicity. Consequently, acellular therapies have garnered interest among researchers. In light of this, the integration of cell sources and exosomes has emerged as a pivotal advancement in addressing the bioactivity limitations of nerve conduits. Numerous experimental studies have shown that nerve conduits combined with stem cells or exosomes yield superior repair outcomes for PNI compared to empty conduits; however, their efficacy still does not match that of the autologous nerve graft cohort [13–15].

Researchers are employing many strategies to augment the efficacy of nerve conduits by enhancing stem cells or exosomes, optimizing nerve conduit function, and seeking to amplify physical stimulation. This article aims to review the current research advancements regarding the application of stem cells and exosomes in conjunction with nerve conduits and to delineate future research directions for the development of nerve conduits.

## Stem Cells

### Definition and classification

Stem cells represent a unique category of cells characterized by their ability to undergo indefinite proliferation and differentiate into all 3 germ layers [16]. Their remarkable self-renewal capacity and potential to differentiate into specialized cell lineages render stem cells a valuable resource in the field of regenerative medicine, particularly for cell replacement therapies [17]. Presently, stem cells utilized in clinical settings are primarily classified into 3 categories: embryonic stem cells (ESCs), adult stem cells (ASCs), and induced pluripotent stem cells (iPSCs). While ESCs exhibit the highest differentiation potential, their clinical application is constrained by ethical considerations and the risk of tumorigenicity. Conversely, ASCs, which possess more limited differentiation capabilities, are more readily accessible. This category includes hematopoietic stem cells, mesenchymal stem cells (MSCs), and neural stem cells, which are regarded as the gold standard in clinical stem cell therapy [18]. Among these, different varieties of MSCs possess distinct characteristics and may substantially contribute to the repair of peripheral nerves [19].

Regarding the ethical issues of ESCs, as early as 1962, Gurdon demonstrated that the artificial method of somatic cell nuclear transfer (SCNT) endows cells with totipotency, paving the way for epigenetic reprogramming [20,21]. In recent decades, advancements in reprogramming technologies have enabled researchers to derive iPSCs from somatic cells, which possess functionalities akin to those of ESCs and have found extensive applications in the field of medicine [22]. Due to the differentiation potential and synthetic nature of iPSCs, they also hold great promise for applications in peripheral nerve repair.

### Mechanisms of stem cells in peripheral nerve repair

**Fig. 1.**
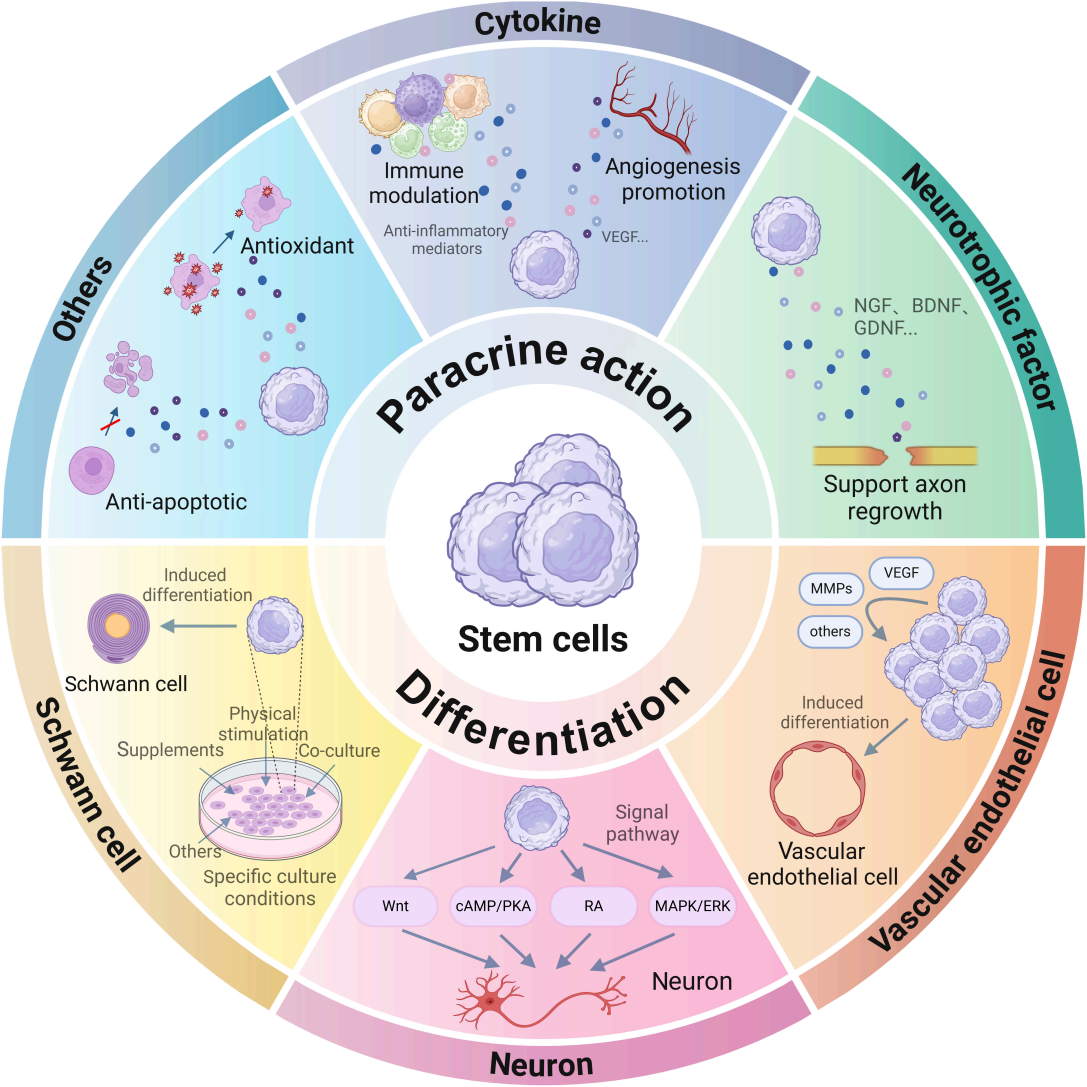
Mechanism of stem cell repair in PNI.

Recent scholarly articles highlight the critical function of stem cells in the repair of nerve injuries, attributed to their autocrine and paracrine activities, as well as their capacity for proliferation, differentiation, and regulation of angiogenesis [23,24]. Furthermore, contemporary research has suggested that stem cells exhibit immune regulatory properties. The following section will provide an overview of the mechanisms underlying stem cell therapy in the context of PNI as shown in Fig. 1 [19,25].

#### Paracrine action

Stem cells release a diverse array of bioactive molecules into the extracellular environment, encompassing cytokines, neurotrophic factors, microRNAs (miRNAs), cell adhesion molecules, proteases, and subcellular organelles. These secretions have the potential to facilitate axonal regeneration, augment angiogenesis, and modulate immune responses [26]. The incorporation of stem cells into nerve conduits can foster an improved microenvironment conducive to peripheral nerve regeneration in applications involving nerve conduits. The subsequent sections will offer a comprehensive overview of the principal secretions of stem cells pertinent to peripheral nerve repair.

Numerous studies have demonstrated that the secretome of stem cells is abundant in neurotrophic factors, which play a crucial role in enhancing myelin thickness, modulating the Wallerian degeneration process, and exhibiting excellent neuroprotective and neurotrophic properties [27,28]. Notably, Salgado and colleagues [29,30] conducted an analysis of the factors secreted by adipose-derived stem cells (ADSCs) across various in vitro microenvironments, identifying 3 primary categories of neurotrophic factors: neurotrophic factors such as nerve growth factor (NGF), brain-derived neurotrophic factor (BDNF), and glial cell line-derived neurotrophic factor (GDNF); additional neurotrophic factors including ciliary neurotrophic factor (CNTF) and leukemia inhibitory factor (LIF); and members of the transforming growth factor-β (TGF-β) family, specifically TGF-β1 and TGF-β2. These factors not only facilitate nerve regeneration but also inhibit neurodegeneration and apoptosis. Concurrently, Lopatina et al. [31] provided evidence through murine experiments indicating that the expression levels of messenger RNA (mRNA) encoding neurophosphatase in human ADSCs (hADSCs) were elevated in vivo, which correlated with an increase in the secretion of neurotrophic factors. The findings from both in vivo and in vitro studies collectively underscore the significance of neurotrophic factor secretion as a critical mechanism through which stem cells mediate peripheral nerve repair.

Stem cells possess the ability to secrete angiogenic factors that facilitate neovascularization, thereby supplying essential oxygen and nutrients necessary for nerve repair. In a study conducted by Kilroy et al. [32], the cytokine profiles of 18 ADSCs donors were analyzed, revealing that ADSCs express a substantial array of angiogenic and hematopoietic factors. Boomsma and Geenen [33] further investigated the effects of conditioned media derived from MSCs in comparison to control media, concluding that specific cytokines secreted by MSCs can enhance angiogenesis. Besides, stem cells also release anti-inflammatory mediators that play a crucial role in modulating inflammation and neuroinflammation [34]. Agarwal et al. [35] demonstrated that nerve conduits infused with bone marrow-derived MSCs (BM-MSCs) can down-regulate chondroitin sulfate 6-sulfate, cyclooxygenase-2 (COX-2), and interleukin-6 (IL-6), thereby inhibiting astrocyte activation, and noted that these cells secrete a substantial quantity of anti-inflammatory factors. Additionally, Chen et al. [36] observed in a rat model of sciatic nerve injury that the administration of ADSCs significantly reduced levels of tumor necrosis factor-α (TNF-α) and IL-1β. Collectively, these findings suggest that stem cells can enhance angiogenesis and modulate immune responses through the secretion of various cytokines, thereby fostering an optimal microenvironment for peripheral nerve repair. Furthermore, existing literature indicates that stem cells exhibit antioxidant [37] and anti-apoptotic [38] properties; however, the specific factors and underlying mechanisms remain to be elucidated.

#### Differentiation

The capacity of stem cells to differentiate into diverse cell lineages constitutes a critical aspect of their therapeutic mechanisms. This differentiation can occur through 2 primary processes: the in vitro directed differentiation of ESCs and iPSCs, and the reprogramming and targeted differentiation of ASCs, with the latter being more commonly utilized in clinical applications [39]. Experimental investigations have demonstrated that stem cells can be directed to differentiate into SCs, neuron-like cells, and vascular endothelial cells, thereby contributing to the replacement of damaged tissue and the establishment of a conducive microenvironment for tissue repair, particularly in the context of peripheral nerve regeneration [25]. The therapeutic potential of stem cells specifically directed to differentiate into these 3 cell types will be discussed in further detail below.

As outlined in the Introduction, SCs are integral to the process of peripheral nerve regeneration, contributing to Wallerian degeneration, axonal regeneration, and myelination. Nevertheless, the direct utilization of SCs for the treatment of PNI presents several challenges, including difficulties in their procurement, slow rates of culture expansion, and elevated immunogenicity [40]. Recent in vitro investigations have indicated that stem cells can be induced to differentiate into SCs. Common methodologies employed in these culture protocols encompass the use of neurotrophic factors (such as NGF, BDNF, and CNTF), small-molecule compounds [including chemical inducers like retinoic acid (RA), β-mercaptoethanol, and dimethyl sulfoxide] [41], gene transfection techniques, and coculture with neuronal cells [42]. Furthermore, external physical stimulation has emerged as a novel strategy for inducing stem cell differentiation. Research conducted by Wu et al. [43] has demonstrated that electrical stimulation (ES) can enhance the differentiation of stem cells into SCs, revealing a synergistic effect when combined with chemical induction (CI) for the targeted differentiation of stem cells into SCs. However, it is important to note that there is currently a lack of in vivo evidence to substantiate the targeted induction of SC differentiation from stem cells.

Researchers have used various chemical substances to influence the differentiation process of stem cells, activating relevant pathways to promote their neural differentiation, mainly through the following pathways: (a) MAPK (mitogen-activated protein kinase)/ERK (extracellular signal-regulated kinase) signaling pathway: Neurotrophic factors bind to membrane surface tyrosine kinase receptors, activating the MAPK/ERK pathway, which in turn regulates transcription factors to induce neural differentiation. It can also activate the phospholipase C–Ca^2+^ (PLC-Ca^2+^) and phosphatidylinositol 3-kinase–Akt (PI3K-Akt) pathways in a similar manner [44]. (b) RA signaling pathway: RA preactivates retinoid signaling, which can improve the neuronal differentiation of MSCs [45]. (c) Cyclic adenosine monophosphate (cAMP) and protein kinase A (PKA) signaling pathway: Hirsutenone (a plant extract) increases the level of cAMP, phosphorylating PKA in MSCs, thereby activating MAPK and promoting neuronal differentiation [46]. (d) Wnt signaling pathway: A biphasic (CHIR-boost) WNT activation strategy can promote the differentiation of iPSCs into mDA neurons [47].

The formation of new blood vessels, or angiogenesis, is a critical process in the repair of peripheral nerves. Stem cells not only secrete factors associated with angiogenesis but also possess the capability to differentiate into endothelial cells, thereby facilitating this process. Almalki et al. [48] demonstrated that the silencing of matrix metalloproteinases MMP-2 and MMP-14 in ADSCs leads to an increased expression of the endothelial cell marker EC and inhibits the cleavage of vascular endothelial growth factor receptor 2 (VEGFR2), thereby promoting the differentiation of ADSCs into endothelial cells. Additionally, Ikhapoh et al. [49] found that angiotensin signaling can enhance the differentiation of MSCs into endothelial cells through the mediation of VEGF-A. Collectively, these findings suggest that stem cells can be effectively induced to differentiate into endothelial cells, which is advantageous for the regeneration of peripheral nerves.

## Exosomes

Numerous experimental investigations have demonstrated that paracrine signaling serves as a fundamental mechanism through which stem cells facilitate neurorepair [50]. Some researchers have inferred from human skeletal muscle-derived multipotent stem cell (Sk-MSC) transplantation studies that approximately 60% to 80% of nerve damage repair within a 12-week period can be ascribed to paracrine effects [51]. The bioactive molecules previously mentioned are primarily secreted extracellularly via extracellular vesicles (EVs) and cell membrane pathways. Notably, EVs are nanoscale particles released by stem cells that encapsulate proteins, RNA, and other biomolecules, including subcellular organelles, which they transport to adjacent or distant cells to exert their biological effects [52,53]. EVs are recognized for their potent metabolic regulatory functions, and employing them to influence the metabolic conditions of diseases like obesity and type 2 diabetes is deemed highly promising [54]. Furthermore, EVs are utilized in the treatment of neurodegenerative disorders, including Parkinson’s disease [55]. Recent studies on EV-based immunotherapy have highlighted the paracrine mechanisms of stem cells, especially the secretion processes via EVs, as a focal point of investigation. EVs generated from stem cells, particularly those from MSCs, have emerged as a prospective study focus due to their dual functions in tissue repair and immunological modulation [56].

**Fig. 2.**
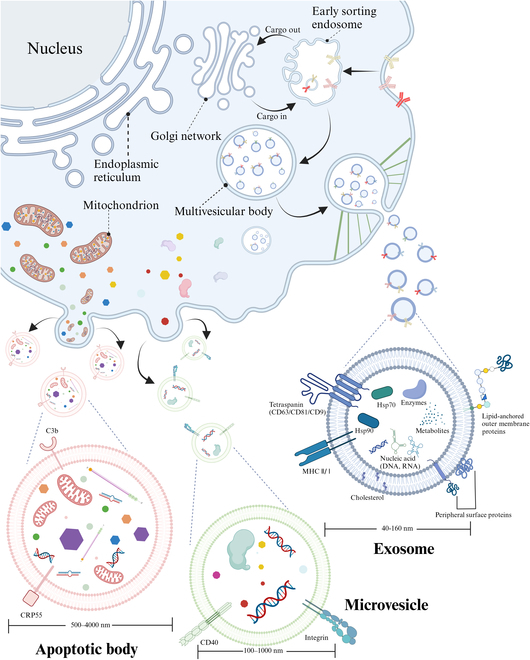
Biogenesis and structure of exosomes, and the comparison between autophagosomes, microvesicles, and exosomes.

EVs encompass exosomes, microvesicles, and apoptotic bodies, with the comparison between them presented in Fig. 2. Apoptotic bodies, ranging from 0.5 to 4 μm, are generated randomly from the plasma membrane during the process of cell apoptosis. In contrast, microvesicles, which measure between 0.1 and 1 μm, are formed through direct budding and secretion from the plasma membrane. Exosomes, the smallest of these vesicles at 40 to 160 nm, are produced via the endosomal pathway. Research has demonstrated that exosomes serve as the primary vesicular mechanism for paracrine signaling within cells [57]. Moreover, the specific proteins contained within exosomes are crucial for mediating anti-inflammatory responses and regulating immune functions [58]. Consequently, exosome therapy represents a novel strategy for mitigating immune rejection and tumorigenicity in the context of stem cell therapy. In studies on rheumatoid arthritis treatment, there is great interest in utilizing therapeutic exosomes as an alternative to cell-based therapies to address limitations such as storage, immunogenicity, and challenges in adhering to good manufacturing procedures [59]. Utilizing exosomes to influence the behavior of fibroblast-like synoviocytes is regarded as a promising approach [59]. Moreover, the application of artificially produced novel pharmaceuticals administered through exosomes to target regions of nerve injury has emerged as a potential technique to augment the efficacy of PNI treatment.

### Structure

Exosomes are EVs characterized by a diameter ranging from 40 to 160 nm, with an average size of approximately 100 nm [60]. These vesicles are secreted by nearly all cell types, including MSCs, immune cells, neurons, cancer cells, epithelial cells, osteocytes, and myocytes [57]. Exosomes are abundantly found in various biological fluids, including urine, serum, plasma, lymph, cerebrospinal fluid, semen, amniotic fluid, blood, and breast milk [60,61]. They are integral to numerous physiological processes, such as cellular communication, immune modulation, reproduction, and development in mammals, as well as neural signaling [61,62]. Furthermore, exosomes are implicated in the pathogenesis of several conditions, including cancer, neurodegenerative disorders, metabolic diseases, cardiovascular diseases, and viral infections [61].

Figure 2 illustrates the structural composition of exosomes, which are characterized by a substantial abundance of surface proteins. Additionally, the internal lumen of exosomes encompasses a variety of biomolecules, including proteins, DNA, RNA, amino acids, and metabolites [61].

Mass spectrometry analysis indicates that the concentrations of cholesterol, sphingomyelin (SM), glycosphingolipids, and phosphatidylserine (PS) in exosomes are 2 to 3 times greater than those found in cells [63]. The substantial enrichment of PS offers novel insights into the methodologies for exosome separation. These enriched lipids may play a crucial role in the biogenesis of exosomes. For instance, the exosome formation process shares several characteristics with microautophagy; the molecular chaperone HSC70 is known to bind to PS, which is specifically associated with the microautophagy pathway. Consequently, the elevated levels of PS may be closely linked to the biogenesis of exosomes. Furthermore, the involvement of cholesterol in the processes related to multivesicular bodies (MVBs), intraluminal vesicles (ILVs), and exosome biogenesis has been the subject of extensive research [64–66].

Research indicates that approximately 25% of human proteins are capable of being secreted via exosomes [67]. They are primarily sorted into exosomes through the ESCRT mechanism and non-ESCRT-dependent pathways (such as proteins containing the KFERQ motif pentapeptide in a manner dependent on the membrane protein LAMP2A [68]). These proteins can be classified into 3 principal categories: transmembrane proteins, lipid-anchored proteins, and peripheral membrane proteins located on the membrane surface [69]. Furthermore, the lumen of exosomes contains a substantial quantity of soluble proteins, and the diversity in exosomal composition suggests that the types of soluble proteins present in the lumen may be influenced by the specific cell lines from which the exosomes are derived.

Transmembrane proteins predominantly consist of 4 key proteins: CD9, CD63, CD81, and CD82, along with CD106, Tspan8, and intercellular adhesion molecule-1 (ICAM-1) [60]. These proteins are highly prevalent on the surface of exosomes, with CD81 being the most abundant, followed closely by CD63. CD81, CD63, and CD9 are extensively utilized as biological markers for exosomes [69]. Additionally, CD9 and CD81, in conjunction with ICAM-1, have been demonstrated to facilitate the uptake of exosomes [70,71]. Certain transmembrane proteins, such as CD63 and syndecans, are incorporated into exosomes through a mechanism regulated by heparinase, which involves the syntenin–ALIX–ESCRT pathway [72].

Lipid-anchored proteins are categorized into 2 groups: lipid-anchored internal proteins and lipid-anchored external proteins. The latter includes various glycosylphosphatidylinositol (GPI)-anchored proteins, such as CD39, CD73, the sperm receptor Juno, CD55, and CD59, as well as Hedgehog morphogens. Lipid-anchored internal proteins encompass guanosine triphosphatases (GTPases) (including Rabs, Ras, and Rho) and myristoylated signaling kinases, such as Src, among others [69].

Research indicates that exosomes are capable of transporting external peripheral proteins, which include TGF, TNF, TNF-related apoptosis-inducing ligand, Fas ligand, cytokines, and a variety of other surface signaling molecules that participate in the transduction of multiple signaling pathways [69]. Internal peripheral membrane proteins encompass a range of cytoskeletal proteins, such as ERM proteins (including EBP50, CD44, CD43, IGSF8, and PTGFRN) [69]. The tumor susceptibility gene 101 protein (TSG101), classified as a cytoskeletal protein, has been utilized as a marker for exosomes in experimental settings [73,74]. Furthermore, heat shock proteins (HSPs) also represent a category of internal peripheral membrane proteins, functioning as molecular chaperones and exhibiting anti-apoptotic properties in tumor contexts [60]. Experimental findings have demonstrated that exosomes derived from tumors in murine models present tumor antigens, specifically HSP (HSP70-80) and major histocompatibility complex class I (MHC-I) molecules, to dendritic cells (DCs), thereby eliciting a robust CD8^+^ T cell-dependent antitumor response [75].

Exosomes are known to harbor various forms of DNA, including single-stranded DNA, genomic DNA, mitochondrial DNA, and reverse transcription complementary DNA [69]. Studies have shown that exosomes do not contain double-stranded DNA (dsDNA) or histones [76]. Additionally, the precise distribution of DNA within exosomes—specifically, the proportion that resides within organelles versus that which is associated with the surface—remains ambiguous [69]. There exists a lack of consensus in the literature concerning the mechanisms involved in the sorting of DNA into exosomes [77].

Exosomes are vesicles that encapsulate RNA and possess the capability to transfer these extracellular RNAs (exRNAs) to various cells and tissues in a biologically active form. They are particularly abundant in small noncoding RNAs (ncRNAs), which include small nuclear RNAs (snRNAs), miRNAs, transfer RNAs (tRNAs), Y RNAs, vault RNAs, repetitive element RNAs, and fragmented RNAs [69].

Extensive research has been conducted on the role of these RNAs in cancer. Studies have demonstrated that snRNAs derived from exosomes in lung cancer or melanoma can facilitate the establishment of a premetastatic microenvironment by activating Toll-like receptor 3 (TLR3) and promoting the release of cytokines. Additionally, exosome-derived miR-122 has been shown to enhance nutrient availability in the premetastatic microenvironment of cancer cells, thereby supporting metastatic processes [60]. Beyond cancer, exosomal miRNAs play crucial regulatory roles in insulin resistance and pancreatic β-cell dysfunction, both of which are associated with the development of diabetes mellitus [78]. Moreover, mRNA encoding the zinc finger protein ZFY, identified in amniotic fluid exosomes, can serve as a biomarker for determining fetal sex [62]. For insights into the mechanisms underlying RNA sorting within exosomes, one may consult the research conducted by Han et al. [77].

Exosomes demonstrate great variety, seen in their dimensions and composition. The heterogeneous sizes of exosomes are probably attributable to uneven invagination of the limiting membrane of the MVB or the mixture of additional EVs during the isolation procedure [61]. This size heterogeneity may result in discrepancies in the quantity of substances within the exosomes. The variance in contents is presumably associated with particular sorting systems. Nevertheless, as of now, aside from a limited number of pivotal proteins like CD63 [72], syndecans [72], and Hsp90α [79], whose incorporation into exosomes is comprehensively recognized, the sorting mechanisms for other cargo molecules have yet to be clarified. The ambiguous sorting mechanisms exacerbate the challenge of addressing exosome variability.

### Biogenesis

The classical process of exosome generation comprises 4 distinct stages, as illustrated in Fig. 2. First, the invagination of the plasma membrane leads to the formation of early secretory endosomes. Second, following the transport of cargo, inward budding takes place, resulting in the creation of ILVs within the endosome, a process commonly referred to as the formation of MVBs. Third, late endosomes undergo maturation through acidification. Finally, MVBs merge with the cell membrane, facilitating the release of ILVs as exosomes into the extracellular environment [60].

The exosome release facilitated by SNARE complexes is regarded as the conventional mechanism for completely mature MVBs to merge with the plasma membrane, hence discharging exosomes extracellularly. Research indicates that particular SNARE protein variants can affect the secretion of various exosome types [80]. Rab proteins participate in the assembly of SNARE complexes, and research has demonstrated that GTPase-activating proteins (TBC1D10A–C) specifically target Rab35 to modulate exosome secretion in an activity-dependent fashion [81]. Moreover, cellular retinoic acid binding protein 1 (Crabp1) has been established as a negative regulator of neuronal exosomes [82]. Rapamycin complex 1 (mTORC1) can likewise inhibit the release of exosomes [83].

### The mechanism of miRNA delivery via exosomes in PNI repair

In the context of exosomes and their involvement in PNI, the mechanisms associated with vascular regeneration, inflammation modulation, SC activation, and axonal regeneration have been extensively discussed in the literature, particularly in the works of Yu and colleagues [57,84]. Additionally, the significance of miRNAs contained within exosomes in the process of peripheral nerve regeneration has been highlighted in various reviews [85]. Recent years have witnessed a surge in research focusing on the role of miRNAs in the repair of PNI. This article seeks to synthesize the recent advancements regarding the applications and mechanisms of miRNAs in the context of PNI, thereby augmenting existing knowledge.

With respect to immune regulation, findings indicate that the expression of miR-146a-5p, which is derived from SC-derived exosomes (SC-Exos), is significantly diminished in the ischemic and hypoxic microenvironment characteristic of PNI. This reduction leads to a notable decrease in its capacity to inhibit inflammation-related damage mediated by the TRAF6/nuclear factor κB (NF-κB) signaling pathway, resulting in a shift of macrophages from the M2 phenotype to the M1 phenotype [86]. The M1 and M2 macrophages are known to play opposing roles in the modulation of inflammation, with M1 macrophages promoting and M2 macrophages inhibiting inflammatory processes. In a study conducted by Fan et al. [87], exosomes (Exo-146a) derived from bone marrow mesenchymal cells that overexpress miR-146a were utilized. The results demonstrated that Exo-146a facilitated a more rapid improvement in peripheral nerve function compared to exosomes (exo-naïve) lacking miR-146a overexpression, while also significantly inhibiting the transition of macrophages to the M1 phenotype. Furthermore, the miR-146a present in these exosomes targets the gene IRAK1, thereby enhancing the suppression of TLR4, MyD88, and NF-κB expression, which in turn reduces the secretion of pro-inflammatory cytokines such as TNFα and IL-1β [87].

The development of SCs is characterized by a series of transitional stages. Initially, SC precursors (SCPs) undergo rapid proliferation, leading to the formation of immature SCs. Subsequently, these immature SCs differentiate into either myelinating SCs or nonmyelinating/Remak SCs [88]. In response to PNI, both myelinating and nonmyelinating/Remak SCs engage in adaptive reprogramming, which entails the down-regulation of myelin transcription factors and the up-regulation of various immature SC markers. This process ultimately results in the transformation of myelinating and nonmyelinating/Remak SCs into repair SCs [89]. Following the completion of axonal regeneration, repair SCs can further differentiate back into myelinating SCs or nonmyelinating/Remak SCs [90]. Recent studies have demonstrated that exosomes derived from endothelial cells can enhance and sustain the repair-related phenotype of SCs [91]. Animal model validations have shown that these endothelial cell-derived exosomes facilitate vascular regeneration, axonal regeneration, and myelination. Experimental findings suggest that these beneficial effects on nerve injury repair may be linked to a significant up-regulation of miR199-5p expression in SCs, as well as the activation of the PI3K/Akt/PTEN signaling pathway associated with endothelial cell-derived exosomes [91].

Moreover, human adipose-derived MSCs (hADMSCs) possess the capacity to differentiate into an SC phenotype, referred to as hADMSC-SCs. Research conducted by Liu et al. [92] indicates that exosomes derived from differentiated hADMSCs (dExo) exhibit greater efficacy than those from undifferentiated hADMSCs (uExo) in terms of down-regulating pro-inflammatory gene expression and cytokine secretion, as well as promoting axonal growth in sensory neurons derived from iPSCs. Notably, the miRNA content in dExo is significantly higher than that in uExo, with miRNA-132-3p and miRNA-199b-5p, both associated with inflammatory responses, displaying differential expression between the 2 exosome types [92] Additionally, miRNA-21-5p has also been identified as differentially expressed in both groups, with findings from López-Leal et al. [93] suggesting that miRNA-21 plays a role in promoting axonal growth.

Research conducted by Simeoli et al. [94] has demonstrated that following nerve injury, miR-21-5p is released via exosomes from dorsal root ganglion (DRG) neurons, facilitating the recruitment of inflammatory cells in the aftermath of peripheral nerve damage. Additionally, Sun et al. [95] have shown that hypoxic treatment can enhance the efficacy of exosomes derived from SCs in promoting neurovascular regeneration. This enhancement is attributed to the higher concentration of miR-21-5p in hypoxia-treated SC exosomes (H-SCs-Exos) compared to those from normoxic conditions (N-SCs-Exos). The increased miR-21-5p content augments endothelial cell glycolysis by targeting and promoting hypoxia-inducible factor-1α-induced glycolysis while simultaneously inhibiting PDH-E1α-mediated oxidative phosphorylation, thereby facilitating neurovascular regeneration [95]. Furthermore, investigations into paclitaxel (PTX)-induced peripheral neuropathy (PIPN) have revealed that SC-EXOs exert a great protective effect on DRG cells subjected to PTX damage. Mechanistic studies suggest that SC-EXOs ameliorate peripheral neuropathy by up-regulating miR-21, which subsequently mediates the PTEN signaling pathway [96].

Wang et al. [97] utilized hypoxia-preconditioned exosomes derived from bone marrow MSCs (BMSCs) to enhance the proliferation, migration, and paracrine activities of SCs, thereby expediting the repair of facial nerve injuries. Circular RNA (circRNA) analysis revealed that the efficacy of Hypo-Exos is mediated through the circRNA_Nkd2/miR-214-3p/MED19 signaling axis [97]. Additionally, research indicates that exosomes from myeloid-derived suppressor cells (MDSCs) that overexpress miR-214 can stimulate the migration and proliferation of SCs, elevate the expression of neurotrophic factors, and promote the elongation of DRG neuron axons [98]. It is posited that miR-214 activates the Janus kinase 2 (JAK2)/signal transducer and activator of transcription 3 (STAT3) signaling pathway by inhibiting phosphatase and tensin homolog (PTEN) [98]. The research conducted by Zhu et al. [99] on enhancing myocardial repair through exosomes similarly demonstrated that exosomal miR-214-3p targets PTEN, which regulates p-Akt, consequently mitigating myocardial cell damage.

Furthermore, Chai et al.’s investigation [100] into the application of dental pulp stem cell-derived exosomes (DPSC-Exos) for the treatment of sciatic nerve injury in murine models demonstrated that DPSC-Exos can transfer miR-122-5p from DPSCs to SCs, leading to the inhibition of P53 expression and the subsequent suppression of rapamycin-induced autophagy in SCs. In a related study, Yin et al. [101] identified that exosomes derived from ADSCs (ADSC-Exos) are rich in miRNA-26b, and their findings suggest that miRNA-26b mitigates autophagy in damaged SCs by reducing the levels of karyopherin subunit α 2 (Kpna2).

Huang et al. [102] employed viral transfection technology to generate human umbilical vein endothelial cells (HUVECs) that exhibit overexpression of Netrin-1 (NTN1), referred to as NTN1-HUVECs. They subsequently isolated exosomes (NTN1 EC-EXO) that also overexpress NTN1 from these NTN1-HUVECs. In vitro analyses demonstrated that NTN1 EC-EXO significantly enhanced the regenerative capacity of PC12 cells by down-regulating the expression of let7a-5p within these cells.

In the investigation conducted by Liang et al. [103] regarding engineered exosomes for drug delivery, their results indicate that miRNA and small interfering RNA (siRNA) are among the most extensively studied therapeutic agents. The prevalent use of miRNA-based therapies in areas such as oncology suggests a promising potential for their application in the treatment of PNI. However, research by Jeppesen et al. [76] highlights that conventional exosomes are deficient in RNA-binding proteins (RBPs). Furthermore, it has been established that tRNA and the majority of other small ncRNAs (sncRNAs) are susceptible to degradation by ribonucleases (RNases) in the presence of detergents [104]. Consequently, relying solely on exosomes for the delivery of miRNAs may compromise the functional integrity of the miRNAs, despite the protective role of the phospholipid bilayer. This raises concerns regarding the long-term efficacy of miRNAs in the context of PNI treatment. Additionally, the introduction of exogenous miRNAs has the potential to disrupt the expression levels of endogenous miRNAs and mRNA [105]. The interactions among miRNAs and the mechanisms to ensure that exogenous miRNAs does not greatly disrupt the overall equilibrium of intracellular miRNAs and mRNA warrant further investigation [106]. Typically, the dosage of miRNA therapy exceeds the physiological range of endogenous miRNA expression, which may result in unpredictable off-target effects [107]. Therefore, accurately determining the therapeutic dosage of miRNAs is a critical consideration for the advancement of exosome-delivered miRNA therapies into clinical applications.

## Advancements in the Clinical Utilization of Stem Cells and Exosomes in Enhancing Nerve Conduit Development

**Fig. 3.**
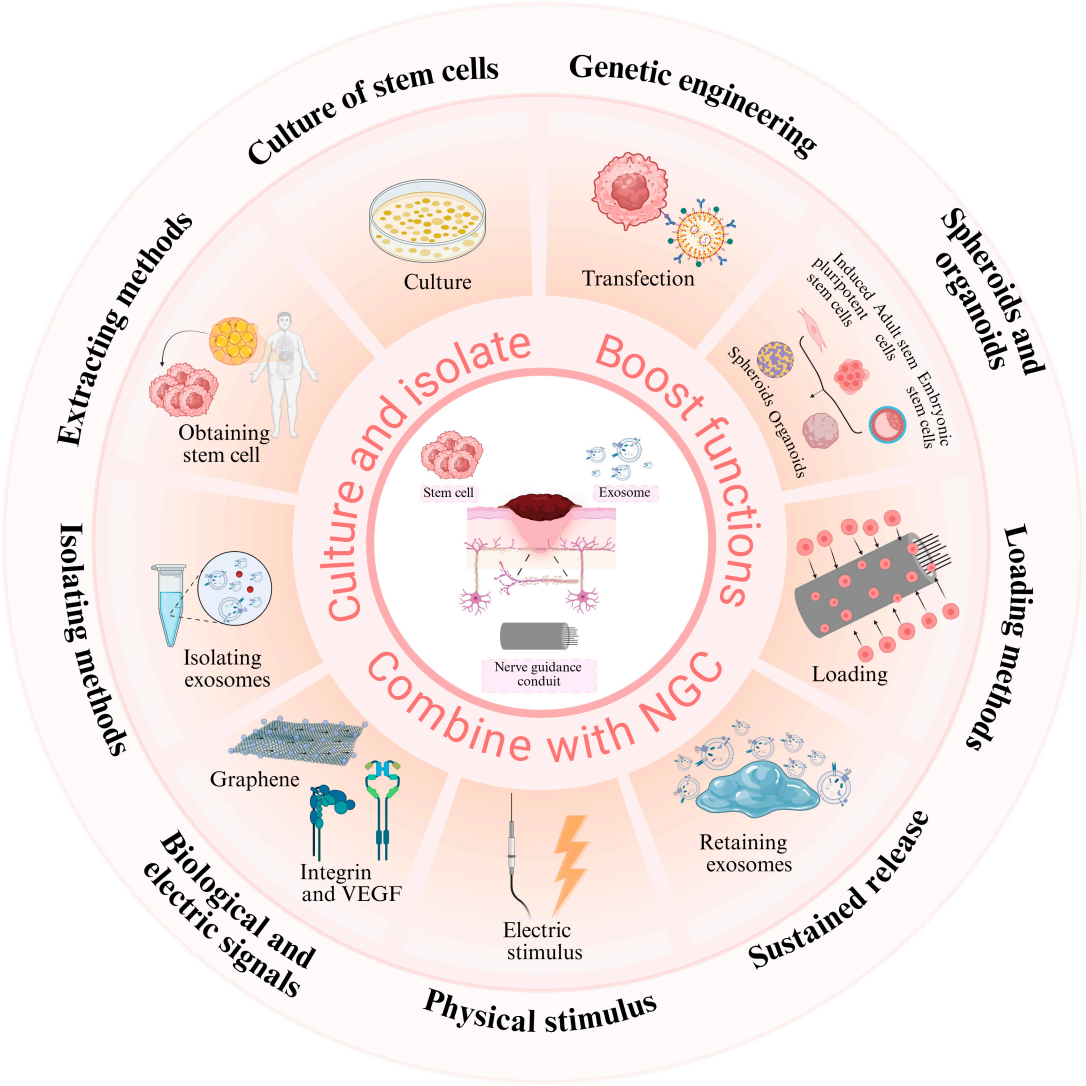
Research progress of stem cells and exosomes combined with nerve conduits.

Despite the extensive utilization of stem cells in tissue engineering, their integration with nerve conduits for peripheral nerve repair presents several limitations. For instance, there are no straightforward techniques to acquire bulk stem cells, the survival rate of stem cells incorporated into nerve conduits is poor, and the precision of targeting stem cell neural differentiation is inadequate. The aforementioned deficiencies have hindered the successful implementation of stem cells in nerve conduits from attaining the anticipated outcomes. Despite numerous advantages of exosomes including the absence of immune rejection, the transition of exosomes into clinical practice is impeded by challenges such as the difficulty in efficiently isolating high-purity exosomes and determining appropriate dosages. Moreover, the low retention and high clearance rates of exosomes are critical factors that compromise the stability and sustainability of their therapeutic efficacy. Additionally, both natural stem cells and exosomes may exhibit insufficient effectiveness, prompting research into the pretreatment of these entities as a promising area of investigation. This article intends to offer a thorough review of recent breakthroughs in the application of stem cells and exosomes to enhance the efficiency of peripheral nerve repair in conjunction with nerve conduits, as shown in Fig. 3.

### Optimizing the source and function of stem cells

Advancements in stem cell research predominantly concentrate on their origins and functionalities. The origin of stem cells pertains to the specific categories of stem cells chosen and the techniques employed for their isolation. About the acquisition of stem cells, BMSCs and human iPSCs are frequently utilized as stem cell source from a sourcing perspective; however, the acquisition of these cells is often associated with painful procedures. Consequently, researchers have turned their attention to adipose tissue, which is known to contain a higher concentration of stem cells. Within this context, 2 notable types of stem cells have emerged: ADSCs and dedifferentiated fat (DFAT) cells. Both types offer several advantages, including abundant availability, ease of handling, and rapid proliferation rates, and they have been shown to possess comparable or even superior properties relative to BMSCs [108,109]. Furthermore, ongoing research is investigating additional stem cell sources for peripheral nerve repair, each presenting unique benefits to address diverse clinical requirements. These sources include DPSCs, peripheral blood MSCs (PBMSCs), and multipotent vascular stem cells (MVSCs). Notably, DPSCs, which are derived from ectodermal neural crest cells, demonstrate a greater capacity for nerve and blood vessel generation compared to mesoderm-derived stem cells such as BMSCs and ADSCs [110]. PBMSCs, which are obtained from bone marrow, exhibit differentiation potential akin to that of BMSCs, and their low-cost, noninvasive acquisition method has garnered great interest among researchers [111]. Additionally, MVSCs possess a heightened ability to differentiate into perivascular cells, which play a crucial role in supporting and stabilizing newly formed microvessels, thereby facilitating peripheral nerve regeneration [112].

Researchers have made marked advancements in the extraction of ADSCs from adipose tissue, which is promising. The conventional enzymatic digestion technique poses a risk of introducing xenogeneic components that may elicit immune responses, thereby constraining its clinical applicability. Consequently, there has been a focus on enzyme-free mechanical separation techniques. Sherman et al. [113] introduced an explant method that entails segmenting adipose tissue into fragments smaller than 1 mm for culture, which successfully recovers ADSCs; however, this method is characterized by a low recovery rate and an extended acquisition period. Conversely, Sawai et al. [114] effectively micronized adipose tissue utilizing the Adinizer fat tissue micronizer, resulting in an enriched yield of ADSCs while maintaining a greater proportion of the extracellular matrix (ECM), which is advantageous for the survival of ADSCs. In vivo studies have demonstrated that the ADSCs obtained through micronization method exhibit comparable effectiveness to those derived from the enzymatic digestion technique.

**Fig. 4.**
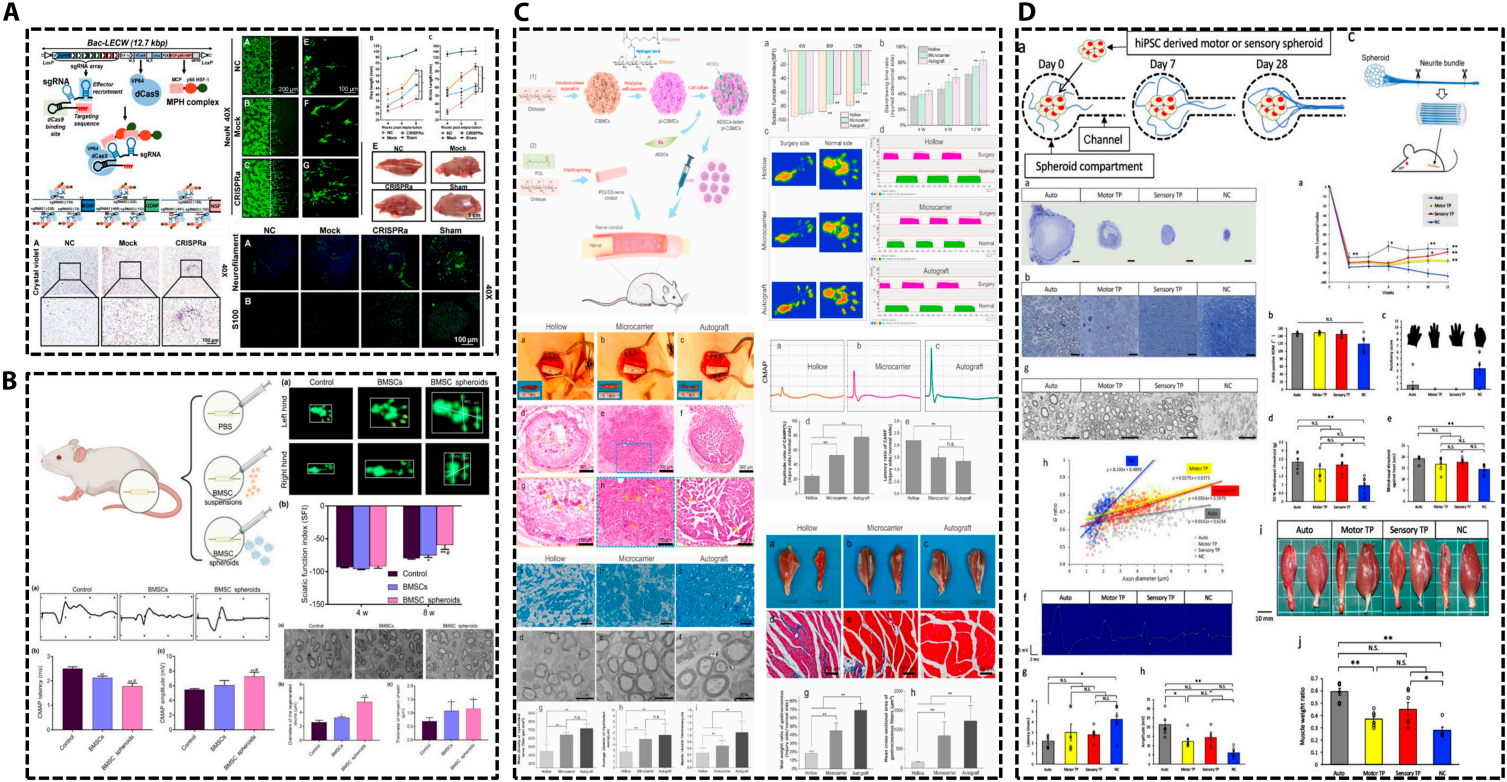
Improved stem cell function promotes peripheral nerve repair. (A) Gene modification of stem cells expressing BDNF, GDNF, and NGF using the CRISPRa system. Reproduced with permission from [119]. Copyright 2019, Theranostics. (B) 3D culture scheme forming BMSC spheroids. Reproduced with permission from [137]. Copyright 2021, Polymers. (C) Chitosan microcarriers modified with poly-l-lysine loaded with stem cells. Reproduced with permission from [143]. Copyright 2021, Bioactive Materials. (D) Artificial nerves induced from iPSCs. Reproduced with permission from [152]. Copyright 2024, Inflammation and Regeneration.

In addition to the source of stem cells, researchers are increasingly prioritizing the enhancement of stem cell functions to attain favorable outcomes, as illustrated in Fig. 4. These outcomes encompass gene modification, specialized cultivation methods, loading techniques, and the in vitro induction of stem cells to develop organoids, which are utilized in the repair of peripheral nerves.

#### Modification and cultivation of stem cells

As previously noted, neurotrophic factors are crucial in the regeneration of peripheral nerves, serving as effective paracrine agents of stem cells. Consequently, the application of stem cells that exhibit overexpression of neurotrophic factors has emerged as a vital research strategy aimed at enhancing the functional characteristics of these cells. Over a decade ago, research teams led by Shi et al. [115] and Fu et al. [116] demonstrated through gene transfection that stem cells with elevated levels of GDNF and BDNF exhibited superior repair capabilities when utilized in nerve conduit applications. Furthermore, Zhang et al. [117] employed lentiviral cotransfection of GDNF and BDNF, establishing that a dual-factor strategy yielded more effective outcomes compared to a single-factor approach. However, traditional plasmid transfection is characterized by low efficiency, and lentiviral transfection is constrained by packaging capacity limitations. Recent advancements in gene transfection technologies have addressed these challenges. Hsu et al. [118] developed a transfection methodology utilizing a Cre/loxP hybrid baculovirus (BV) system, which significantly enhanced transduction efficiency to 97%. They subsequently integrated a BV virus with a CRISPR activation (CRISPRa) delivery system to further improve packaging capacity, successfully cotransfecting BDNF, GDNF, and NGF to achieve sustained gene overexpression for over 21 days [119]. In addition to the introduction of neurotrophic factor genes via gene engineering techniques and the subsequent induction of their overexpression, researchers are increasingly directing their attention toward cells that naturally exhibit elevated levels of neurotrophic factors within the organism. A notable example is the investigation conducted by the Ahmed team, which examined the endogenous expression of neurotrophic factors in DPSCs. Their findings revealed that the fibroblast growth factor 2 (FGF2) significantly enhances the endogenous expression of neurotrophic factors in decellularized peripheral nerve conduits (DPCs). The efficacy of this enhancement in facilitating peripheral nerve repair was substantiated through both in vivo and in vitro experimental methodologies [120].

The isolated stem cells must initially undergo in vitro culture and expansion prior to their incorporation into the nerve conduit. Consequently, the conditions of the culture environment will play a crucial role in influencing the viability and characteristics of the stem cells.

The selection of an appropriate culture medium for stem cells is a critical consideration in cell culture practices. Fetal bovine serum (FBS) is commonly utilized as a growth factor supplement; however, its use presents several drawbacks, including the potential to provoke immune responses and an increased risk of contamination from viruses, bacteria, and prions [113]. Furthermore, the quality of FBS can exhibit variability across different batches, which can compromise the reliability and reproducibility of experimental outcomes. Consequently, there has been a growing emphasis on identifying supplements that are devoid of xenogeneic protein components, are easily extractable, and possess more consistent definitions in the context of stem cell culture [121]. Recent investigations have indicated that human platelet lysate (hPL) serves as a viable alternative to FBS, offering a culture method that is entirely free of xenogeneic materials. hPL can be sourced in substantial quantities from the buffy coat through sedimentation and has been shown to enhance the status and functionality of stem cells [122]. Research findings suggest that hPL culture significantly elevates the secretion of neurotrophic factors from stem cells, with the levels of 3 principal neurotrophic factors—GDNF, NGF, and BDNF—exceeding those observed in the FBS group by more than threefold. Additionally, hPL demonstrates superior cell stability and proliferation capabilities, and experimental evidence indicates that stem cells cultured in hPL can facilitate neurite outgrowth in coculture systems, thereby yielding improved outcomes in nerve repair [123–125].

The second aspect pertains to the incorporation of cultural substances. As previously noted, the mechanism by which stem cells operate can facilitate their differentiation into SCs through the introduction of neurotrophic factors. Given the challenges associated with the procurement and synthesis of these factors, Omar’s research team [126,127] identified that the traditional herbal remedy *Centella asiatica*, recognized for its use as a nerve supplement, contains extracts that are noncytotoxic and capable of promoting the differentiation of MSCs into Schwann-like cells. Their findings indicated that when these treated cells were combined with decellularized nerve conduits derived from human umbilical cord (HUC), they exhibited effects comparable to those observed with autologous nerve grafting. Additionally, Huang et al. [128] demonstrated that FGF can stimulate neuronal differentiation in ESCs and iPSCs. They identified FGF9 as a critical factor that can induce ADSCs to differentiate into SCs via the FGF receptor 2–Akt signaling pathway. Expanding upon this research, Koh et al. [129] introduced a chemical mixture (VCRFSGY: 0.5 mM valproic acid, 3 μM CHIR99021, 1 μM Repsox, 10 μM forskolin, 10 μM SP600125, 5 μM GO6983, 5 μM Y-27632) into a nerve induction medium containing FGF, which resulted in enhanced repair of motor function in damaged nerve fibers.

The coculture strategy involving stem cells is a prevalent methodology in the field of tissue engineering, wherein various cell types are cultured alongside stem cells to preserve their stemness and facilitate their directed differentiation into specific lineages [130]. Previous studies have indicated that coculturing stem cells with midbrain cells, neurons, and other neural tissues can enhance their differentiation into neural cell types [131,132]. However, the potential application of these combined neural conduits in the context of peripheral nerve repair remains unexplored. In a study conducted by Clark et al. [133], SCs were cocultured with iPSCs, revealing that SCs can enhance the myelination of iPSC-derived sensory neurons via the NRG1-ErbB signaling pathway. Furthermore, Chen et al. [42] developed collagen/alginate neural conduits embedded with SCs and cocultured them with Wharton’s jelly MSCs (WJMSCs). Their findings indicated that SCs not only promote the proliferation of WJMSCs but also augment their nerve regeneration capabilities through the secretion of NGF. Additionally, SCs were shown to facilitate the differentiation of WJMSCs through the release of exosomes, underscoring the great potential of neural cell coculture in the integration of stem cells with neural conduits.

Finally, the 3-dimensional (3D) culture of stem cells promotes the formation of spheroids, which demonstrate enhanced cell viability, differentiation potential, ECM secretion capabilities, and improved survival rates compared to single-cell suspensions. Common methodologies for spheroid culture include the hanging drop method, magnetic method, plate method, and microfluidic technology [134,135]. Research conducted by Zhang et al. [136] indicated that spheroids derived from gingival MSCs (GMSCs) using the plate method more effectively induce differentiation into Schwann-like cells and neurons. Similarly, Li et al. [137] utilized the hanging drop method to generate spheroids from BMSCs and observed an increase in the expression of stemness genes, which correlated with enhanced repair outcomes in a rat model of sciatic nerve injury. Furthermore, during the spheroid formation process, the introduction of nanoparticles or gene delivery systems can be employed. Tseng and Hsu [138] successfully transfected BDNF into MSC spheroids, resulting in improved nerve repair outcomes. Additionally, spheroids facilitate various forms of cellular hybridization. Song et al. [139] constructed hybrid spheroids comprising human iPSCs and human MSCs, revealing that the hybridization of distinct cell types enhances their respective functions, as evidenced by increased neural differentiation.

#### Loading and organoid technology of stem cells

The process of loading stem cells onto nerve conduits is a critical component in the application of stem cells in nerve repair. The conventional static inoculation method, which entails the direct injection of a cell suspension into the nerve conduit, presents several challenges, including cell detachment, leakage, low survival rates of the loaded stem cells, and limited stem cell loading capacity. To address these issues, researchers have transitioned to a dynamic inoculation approach, which supersedes the traditional static method. This innovative technique employs a rotating cell culture system that facilitates both rotation and oscillation during the inoculation period, resulting in a more homogeneous distribution of stem cells and a marked improvement in their survival and proliferation on the nerve conduits [140–142]. To further enhance the loading capacity of stem cells, Sun et al. [143] implemented the use of microcarriers for stem cell delivery, specifically utilizing macroporous chitosan microcarriers (CSMCs) to significantly increase the specific surface area and loading capacity. Additionally, they modified the microcarriers with poly-l-lysine to augment their affinity for cells, thereby improving the loading efficiency of stem cells in the context of nerve repair. Similarly, Zhu et al. [144] reported comparable outcomes by employing CSMCs for the loading of ADSCs.

Furthermore, to replicate the microenvironment conducive to nerve repair within the body and to establish directional gradients of factors that facilitate SC migration, Sun et al. [145] employed photocrosslinking technology to manipulate the resuspension and distribution of cells, encapsulating them in gelatin methacrylate (GelMA). Their findings indicated that the gradient of secreted neurotrophic factors effectively promoted the migration of SCs. In a related study, Liu et al. [146] utilized cell-laden bioink for 3D bioprinting, enabling precise customization of both the quantity and spatial distribution of stem cells within nerve conduits. This approach facilitated the embedding of live cells within the nerve conduit structure, thereby enhancing cell adhesion and functionality. Building upon this foundation, Das et al. [147] integrated carbon nanotubes (CNTs) into the bioink to augment its electroactivity, enhance cell viability, and create a microenvironment that emulates the natural electrical signal transmission characteristic of the nervous system, ultimately fostering nerve repair.

Organoids are synthetic grafts created by disrupting the symmetry of early homogeneous stem cell populations, such as stem cell spheroids. In comparison to stem cells, organoids display biomimetic cellular architectures and replicate the extracellular environment at both macro and micro levels. Research indicates that organ-specific structural transplants yield superior outcomes in nerve regeneration [148]. Currently, the more advanced techniques for generating stem cell-derived organoids encompass 4 primary methods: micropore-based approaches, microfluidic systems, bioreactor technologies, and hydrogel matrices, which have demonstrated specific applications in the repair of bone, intestinal, and hepatic tissues [134]. Nevertheless, the utilization of organoids in the context of peripheral nerve repair remains inadequate.

In their research, Zhang et al. [149] employed cell self-aggregation techniques to create micro-tissues and subsequently assessed the ECM components, including laminin and fibrin, through immunofluorescence analysis. Their findings indicated the effective reparative capabilities of these micro-tissues, as demonstrated in both in vitro coculture and in vivo experimental models. In contrast to the nonspecific approach to micro-tissue construction utilized in this study, an alternative strategy focuses on the targeted development of organoid-like structures derived from stem cells, specifically artificial nerves. Kawada et al. [150] investigated methodologies for the precise induction of stem cells in vitro to differentiate into nerve bundles, particularly by converting iPSCs into spheroids and subsequently transferring them into motor neuron culture media enriched with RA and smoothened agonist (SAG) to facilitate axon bundle formation. Osaki et al. [151] further refined this culture protocol by incorporating additional accelerating agents (SU5402 and DAPT) to enhance the rate of axon bundle development. Building upon this foundation, Nishijima et al. [152] validated through in vivo studies that the transplantation of artificial nerve bundles can facilitate nerve regeneration by activating macrophages and up-regulating genes associated with nerve repair, with anticipated efficacy exceeding that of autologous nerve grafts. Nonetheless, there exists a divergence of opinion regarding the relative reparative effects of sensory artificial nerves compared to motor artificial nerves, and further investigation is warranted to determine whether these 2 types can synergistically enhance overall nerve repair outcomes.

### Optimizing the source and function of exosomes

Exosomes obtained from diverse cell or tissue sources are utilized directly, without further processing, for the repair of peripheral nerve injuries (PNIs), as indicated in Table S1. Table S1 illustrates the functions of exosomes at the tissue or cellular level, along with their potential active components. Exosomes derived from MSCs have gained great attention; however, exosomes from various other tissues or cells also play important roles. Efforts have been undertaken to integrate exosomes derived from MSCs and various tissue sources. Zhang et al. [153] utilized exosomes from platelet-rich plasma to augment the capacity of MSCs in facilitating nerve regeneration.

**Fig. 5.**
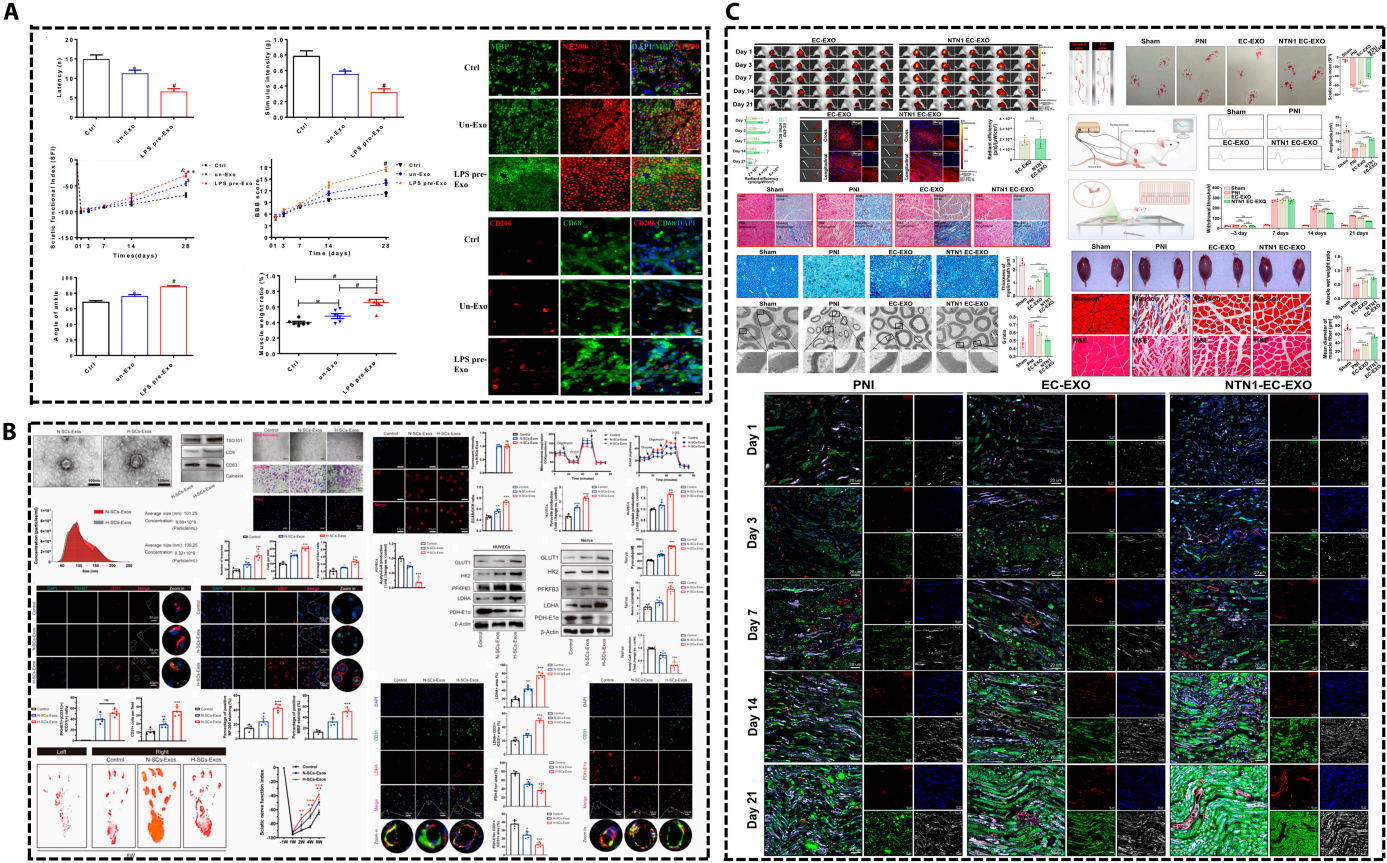
Enhancing exosome function to improve the efficacy of treating PNI. (A) Exosomes derived from LPS-preconditioned BMSCs. Reproduced with permission from [172]. Copyright 2022, Elsevier. (B) Hypoxic SC-derived exosomes. Reproduced with permission from [95]. Copyright 2024, Springer Nature. (C) NTN1-engineered endothelial cell exosomes. Reproduced with permission from [102]. Copyright 2024, American Association for the Advancement of Science.

To enhance the functionality of exosomes, one strategy involves employing genetic engineering and related techniques to induce the overexpression of specific molecules that can positively influence the microenvironment conducive to nerve repair. Alternatively, another strategy entails subjecting the source cells of exosomes to challenging conditions, such as hypoxia and lipopolysaccharide (LPS) stimulation, with the objective of producing exosomes that exhibit improved efficacy in promoting nerve repair. The remarkable neural benefits observed following the application of these 2 methodologies in experimental settings are illustrated in Fig. 5. In addition, the optimization of exosome isolation technology is also an important advance in exosome-based treatment.

#### Isolation of exosomes

Ultracentrifugation (UC) is regarded as the gold standard for the extraction and separation of exosomes and is the most prevalent technique employed in this context [154]. The fundamental principle underlying UC involves the isolation of target components based on their size and density differentials within a solution, rendering it effective for the separation of substantial quantities of components that exhibit great variations in sedimentation coefficients [154]. To enhance the purity of exosomes, density gradient centrifugation is frequently utilized in conjunction with UC [154]. Nevertheless, the UC method is associated with several limitations, including low yield and variability in results, protracted processing times, the requirement for large sample volumes, the necessity for costly laboratory equipment, and elevated technical expertise among personnel [155]. Consequently, the standardization of exosome separation via UC presents considerable challenges, which obviously impedes the clinical application of exosomes [156].

In light of this, researchers have enhanced separation techniques for exosomes, employing strategies that include size-based separation, immunoaffinity capture, and exosome precipitation. Nevertheless, these approaches frequently lead to contamination with other EVs and ECM components [156]. Consequently, the achievement of high-purity exosomes in a high-throughput and efficient manner continues to pose a considerable challenge, impeding the translation of exosome research into clinical applications.

Conventional separation techniques, alongside the previously mentioned UC method, encompass approaches that rely on particle size and precipitation. It is obvious that these conventional procedures frequently do not achieve both elevated recovery rates and high purity simultaneously, with certain methods potentially harming exosomes. In light of these limitations, some researchers have sought to integrate UC and ultrafiltration with size exclusion chromatography to enhance the efficacy of separation processes [157].

Emerging technologies encompass microfluidic systems, magnetic bead-based immunoaffinity capture, nanomaterial-based immunoaffinity capture, and asymmetric flow field-flow fractionation (AF4) methodologies, each characterized by distinct principles, advantages, and limitations in Table S2.

In the context of immuno-capture, there has been a paradigm shift away from the permanent labeling of exosomes, with a focus on recognizing PS on the exosomal membrane rather than proteins such as CD63. This approach employs the Tim4 protein to facilitate the Ca^2+^-dependent capture of exosomes through its binding to PS, followed by the introduction of a Ca^2+^ chelator to gently release the exosomes from the magnetic beads [158]. Furthermore, advancements in material properties have been explored to enhance immuno-capture efficacy; for instance, Cheng et al. [159] utilized spiky immunomagnetic beads to minimize nonspecific adsorption of protein precipitates and larger vesicles. Additionally, Zhang et al. [160] innovated a novel immuno-material by grafting Tim4 antibodies onto an organic framework, which remarkably improved capture efficiency while simultaneously reducing nonspecific adsorption.

Microfluidic technology has garnered great interest due to its inherent simplicity, cost-effectiveness, ease of automation, and capability to manipulate samples with precision at the microscale. Additionally, it offers rapid analysis times and minimizes the consumption of samples and reagents [155], rendering it particularly suitable for clinical applications, notably in personalized medicine [78]. Presently, microfluidic technologies are primarily categorized into 2 distinct types [155]: label-free methods and label-based methods. Label-free methods circumvent the need for exosome labeling, thereby providing considerable advantages in terms of process simplification, time efficiency, and cost reduction, while simultaneously preserving the biological activity of exosomes [155]. Conversely, label-based methods employ antibodies or aptamers to identify proteins on the exosomal surface or utilize Annexin V to detect PS present on exosomes [161–163]. The targeting of PS or the application of aptamers is advantageous as it allows for milder elution conditions, which better safeguard the biological integrity of exosomes [161,164]. Furthermore, label-based techniques not only are applicable for the separation of exosomes but also facilitate their detection. For instance, Zhao et al. [165] implemented a centrifugal microfluidic disc system for the separation of exosomes, integrating it with an aptamer fluorescence detection system, thereby developing a tool that holds potential for early cancer screening applications.

Due to the heterogeneity of exosomes and the choice of culture media, the separation of exosomes with high purity, high throughput, and high recovery rate remains an obvious obstacle to their clinical application. In the future, leveraging the automation and portability of microfluidic technology, combined with methods that facilitate exosome release through immunoaffinity capture to improve purity, may be a promising solution.

#### Pretreatment of cells derived from exosomes

To augment the functionality of exosomes, researchers have explored not only the artificial overexpression of specific substances but also the prestimulation of the originating cells, which can modify the characteristics of the exosomes. The relationship between exosomes and hypoxic conditions has been the subject of extensive investigation within oncology, revealing that hypoxia not only increases the release of exosomes from tumor cells [166] but also alters their properties [167]. A substantial body of evidence supports the notion that exosomes play a role in mediating the effects of hypoxia within the tumor microenvironment [168]. Furthermore, certain studies have demonstrated that exosomes derived from MSCs that have been pretreated with LPS can enhance the expression of anti-inflammatory factors and facilitate the activation of M2 macrophages [169]. Building on these findings, researchers are now focusing on prestimulating the cells that produce exosomes to improve their functionality, with the aim of achieving enhanced reparative outcomes for PNI.

As previously noted, exosomes obtained from hypoxia-preconditioned stem cells (SCs) have been shown to enhance the energy metabolism of endothelial cells, thereby facilitating neurovascular regeneration [95]. In the research conducted by Sun et al. [95], exosomes from hypoxia-preconditioned stem cells (H-SCs-Exos) and normoxia-preconditioned stem cells (N-SCs-Exos) were utilized in a model of sciatic nerve compression injury. The findings revealed that the group treated with H-SCs-Exos exhibited superior outcomes compared to the N-SCs-Exos group, as evidenced by increased expression levels of NF-200 and MBP, as well as improved sciatic functional index (SFI) scores. Additionally, Wang et al. [97] employed exosomes derived from hypoxia-preconditioned BMSCs, which were found to enhance the proliferation, migration, and paracrine effects of SCs, thereby accelerating the repair process of facial nerve injuries. While both studies indicated that hypoxic treatment did not significantly affect the size of the exosomes [95,97], research conducted by Jiang et al. [167] demonstrated that hypoxia not only increased the quantity of exosome release in various tumor types and other diseases but also modified the contents of these exosomes. Prior discussions have highlighted the alterations in exosomal miRNAs resulting from hypoxic conditions. Li et al. [170] reported that hypoxia influenced the proteomic characteristics of DPSC-Exos, with exosomes isolated under hypoxic conditions enhancing the proliferation, migration, and angiogenic capabilities of endothelial cells.

The study conducted by Mayer et al. [171] employed fibrin conduits that were integrated with ADSCs/progenitor cells preconditioned under hypoxic conditions. In a comparison of outcomes between the group undergoing nerve grafting with fibrin conduits containing hypoxia-preconditioned ADSCs/progenitor cells (the ATCH group) and the group receiving nerve grafting with fibrin conduits containing normoxia-preconditioned ADSCs/progenitor cells (the ATCN group), statistically significant differences in the SFI and static sciatic index (SSI) were noted exclusively at the eighth week. No significant differences were noted at the 4-, 12-, and 16-week intervals. Furthermore, histological assessments revealed no statistically significant differences between the ATCH and ATCN groups [171]. Despite the authors’ favorable conclusions, the efficacy of hypoxia-preconditioned stem cell therapy for the treatment of PNI remains a subject of contention based solely on the findings presented.

Li et al. [172] conducted a study in which they utilized LPS to pretreat human BMSCs, subsequently isolating the exosomes derived from this pretreatment (referred to as LPS pre-Exos). In the sciatic nerve compression model, the group treated with LPS pretreated exosomes demonstrated significant enhancement in both sensory and motor functioning of the sciatic nerve compared to the group receiving exosomes without LPS pretreatment [172]. Furthermore, LPS pre-Exos significantly enhanced axonal regeneration and myelin reformation while modulating macrophage polarization toward the M2 phenotype via the TSG-6/NF-κB/NLRP3 signaling pathway [172].

To improve the functionality of exosomes, researchers have utilized gene transfection methods on cells to produce exosomes with overexpressed miRNAs or proteins, resulting in positive outcomes in experimental contexts. The effects and mechanisms associated with miRNAs have been previously detailed; thus, further elaboration is unnecessary. Additionally, the incorporation of proteins or peptides that facilitate nerve repair represents a straightforward strategy.

Following PNI, neurotrophin-3 (NT-3) has been shown to sustain the repair phenotype of SCs via the TrkC/ERK/c-Jun signaling pathway [173]. NT-3 is currently undergoing clinical trials for its potential application in treating Charcot–Marie–Tooth disease type 1A (CMT1A), a demyelinating disorder affecting peripheral nerves [174,175]. NTN1, a neuroguidance protein, serves multiple functions within both the peripheral and central nervous systems [176]. Moreover, an increasing body of research has identified NTN1’s involvement in the pathogenesis of various inflammatory conditions, including ischemia–reperfusion injury, atherosclerosis, and diabetes. NTN1 exerts diverse effects on processes such as angiogenesis, leukocyte migration, and the phenotypic modulation of macrophages, thereby influencing the inflammatory response, with its effects on inflammation being context-dependent [177]. Consequently, both NT-3 and NTN1 present great potential for advancing the repair of PNI.

Yang et al. [73] employed a viral vector to transfect NT-3 cDNA into ADSCs, subsequently isolating exosomes enriched with NT-3 mRNA from these cells (Exo^NT-3^). These Exo^NT-3^ were incorporated into alginate hydrogels to create a nerve conduit (Exo^NT-3-NGC^), thereby enhancing the retention duration of the exosomes [73]. In a model of sciatic nerve defect, Exo^NT-3-NGC^ demonstrated superior efficacy in promoting sciatic nerve function and mitigating gastrocnemius muscle atrophy when compared to exosomes derived from ADSCs that lacked NT-3 mRNA (Exo^empty^) [73].

In a separate study, Huang et al. [102] investigated the application of NTN1 EC-EXO for the treatment of sciatic nerve compression injuries. Experiments conducted by Huang et al. utilizing NTN1 EC-EXO for the treatment of sciatic nerve compression injuries demonstrate that NTN1 EC-EXO exhibits superior neuroaffinity, a thicker myelin sheath, a reduced *G* ratio, an increased number of blood vessels and proliferative cells, and a more pronounced enhancement in sciatic nerve function compared to the EC-EXO group. These characteristics contributed to a more pronounced improvement in sciatic nerve function. This evidence suggests that facilitating vascular regeneration at the site of PNI and enhancing the microenvironment of the affected area represent viable strategies for the development of engineered exosomes.

Wang et al. [178] conducted a study utilizing ADSC-Exos that express miR-218, administered via tail vein injection in conjunction with 3-hydroxybutyrate-co-3-hydroxyvalerate (PHBV) to address sciatic nerve defects. The experimental design included a low carbon dioxide environment aimed at enhancing the functionality of the exosomes. The findings suggested that the low carbon dioxide conditions may promote ADSCs to secrete exosomes of reduced size, which are enriched in miR-218. However, when evaluating the efficacy of the treatment for sciatic nerve injury, no statistically significant differences were observed in functional and histological outcomes between the low CO_2_ treatment group and the control group, with the exception of the cross-sectional area of the gastrocnemius muscle. Furthermore, the absence of a control group for ADSC-Exos in this study precluded the assessment of whether the increased miR-218 content in the exosomes conferred superior reparative effects. In a related investigation, Fan et al. [87] reported that in a murine model of diabetic peripheral neuropathy, treatment with exo-146a resulted in enhanced local blood flow compared to the exo-naïve group, leading to more pronounced improvements in both sensory and motor nerve functions. Additionally, Zeng et al. [98] employed muscle-derived stem cell exosomes (exo-miR-214) overexpressing miR-214 for localized injection. Their results indicated that these exosomes, when compared to those derived from muscle stem cells transfected with a negative control, yielded significantly greater enhancements in SFI, wet weight of the gastrocnemius muscle, axonal regeneration, and myelination in a model of sciatic nerve compression injury.

## Advancements in Nerve Conduits to Enhance the Function of Stem Cells and Exosomes

**Fig. 6.**
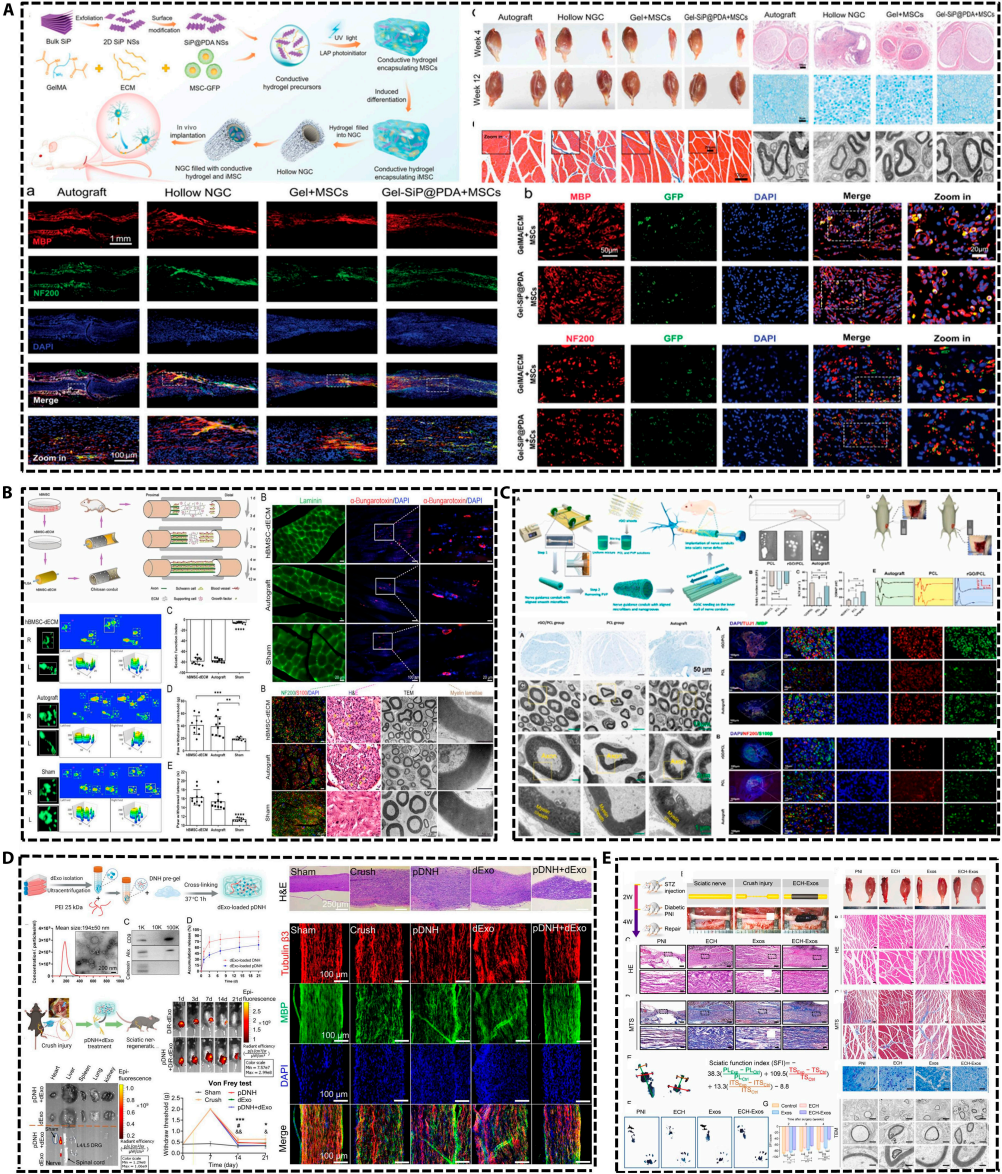
The nerve conduit provides a dynamic specialized microenvironment to promote peripheral nerve repair. (A) Polydopamine-modified silicon phosphate (SiP@PDA) nanosheets incorporated into methacrylated gelatin and decellularized ECM (GelMA/ECM) to construct a conductive hydrogel scaffold. Reproduced with permission from [185]. Copyright 2024, Small. (B) Fabricate bionic 3D cell matrix grafts by using human BMSC-derived decellularized ECM (dECM) combined with PLGA fiber scaffolds. Reproduced with permission from [189]. Copyright 2024, Bioactive Materials. (C) Construction of rGO/PCL nerve conduits containing electrical, biological, and topological cues. Reproduced with permission from [194]. Copyright 2023, Bioactive Materials. (D) Decellularized sciatic nerve hydrogels for sustained release of exosomes. Reproduced with permission from [195]. Copyright 2024, Elsevier. (E) Exosome-loaded electroconductive nerve dressing for sustained release of exosomes. Reproduced with permission from [74]. Copyright 2023, KeAi.

In tissue healing, the biological signals from exosomes or stem cells are crucial, and nerve conduits frequently serve as vehicles for exosomes or stem cells. Nonetheless, the electrical, biochemical, and mechanical signals, together with the sustained release function of exosomes facilitated by nerve conduits, must not be disregarded. Extensive research has been conducted in this area, as illustrated in Fig. 6.

### Optimizing nerve conduit design to enhance electrical signaling, biochemical signaling, and mechanical signals

Stem cells are influenced by a variety of signals emanating from a dynamic and specialized microenvironment referred to as the “niche”. This niche encompasses electrical, biochemical, and mechanical signals [179]. Consequently, effective electrical conductivity is essential for delivering electrical signals, while biochemical signals that mimic the ECM are crucial for facilitating appropriate stem cell functions. Additionally, mechanical signals characterized by specific strengths and guiding cues are vital components in the design of nerve conduits that incorporate stem cell exosomes.

First is the conductive design of the nerve conduit to provide electrical signals. The favorable electrical conductivity properties of nerve conduits mimic the physiological functions of nerves within the body, which is particularly crucial for peripheral nerve repair. Such properties can enhance electrical communication and facilitate the development of stem cells, among other benefits. Several studies have investigated the use of conductive polymers, such as polyaniline (PANi), poly(3,4-ethylenedioxythiophene) (PEDOT), polypyrrole (PPy), and their derivatives, to augment the electrical conductivity of nerve grafts. These studies have confirmed the polymers’ roles in promoting cell adhesion and nerve growth in both in vitro and in vivo settings [180,181]. However, these conductive materials encounter challenges, including significant reductions in conductivity, brittleness, and inadequate processing performance in physiological environments [182]. With the advancement of carbon nanotechnology, researchers have begun to examine the potential of carbon nanomaterials in the development of artificial nerves for nerve conduit fabrication. Multi-walled CNTs (MWCNTs) exhibit exceptional properties, including high strength, flexibility, and conductivity, along with a porous structure that resembles the natural ECM. Liu et al. [183] developed MWCNTs/Co-GelMA hydrogel scaffolds and investigated their efficacy in promoting the differentiation of superior cervical ganglion stem cells (SCAP) into neurons. Their findings indicated that the incorporation of MWCNTs/Co could enhance the neural differentiation of SCAP and exhibited a synergistic effect when combined with ES. Nabipour et al. [184] designed a novel dual-layer polylactic acid/polyurethane/multiwalled carbon nanotube (PLA/PU/MWCNT) nerve guidance conduit embedded with human embryonic neural stem cells (hEnSCs), which demonstrated effective maintenance of cell viability and exhibited pH-regulating anti-inflammatory properties [184]. Xu et al. [185] integrated the benefits of natural ECM and nanotechnology to create a conductive and bioactive nerve conduit by dispersing conductive polydopamine-modified silicon phosphate (SiP@PDA) nanosheets within a GelMA/ECM network. Their results indicated positive outcomes in immune regulation, angiogenesis, and nerve regeneration [185]. These findings suggest that the conductive design of multifunctional hydrogels holds great promise in the realm of nerve repair and may serve as a viable alternative to autologous nerve grafting in the future.

Besides the conductive design, surface modification of nerve conduits is also an important improvement, which simulate ECM to provide biochemical signals. The low survival rate of stem cells presents a great challenge in the application of stem cell therapy for PNI, particularly when used in conjunction with nerve conduits. Consequently, enhancing the surface properties of nerve conduits to replicate the components of the ECM is a critical avenue for improving stem cell viability and functionality. Research conducted by Resch et al. [186] examined the behavior and proliferation of ADSCs and SCs on spider silk substrates, revealing that spider silk facilitates their adhesion, migration, and proliferation. In a separate study, Guiotto et al. [123] utilized animal-derived ECM analogs to culture stem cells on various ECM coatings, discovering that laminin significantly promotes cell proliferation, survival, and functionality through the secretion of neurotrophic factors. Additionally, they combined injectable hydrogels (Biogelx) with neuroactive laminin-derived peptides (IKVAV) to fabricate nerve conduits and investigated their effects on nerve regeneration in vivo. The results indicated positive outcomes across several metrics, although the efficacy remained inferior to that of autologous nerve transplantation [125].

In addition to laminin, the functions of other ECM components, such as fibronectin, remain inadequately understood. In this context, Wang et al. [187] introduced an innovative approach to the surface modification of nerve conduits by employing a decellularization technique to derive ECM. They integrated decellularized porcine peripheral nerve with hydrogel to fabricate nerve conduits, observing that the viability of the encapsulated stem cells exceeded 80%. Similarly, Drewry et al. [188] combined DPCs with naturally aligned ECM, thereby enhancing biocompatibility. Building upon this foundation, Wang et al. [189] developed decellularized ECM (dECM) derived from human BMSCs and combined this dECM with poly(lactic-co-glycolic acid) (PLGA) fiber scaffolds to create a 3D cell matrix nerve graft. In vivo studies demonstrated that this nerve conduit exhibited effects comparable to those of autologous nerve grafting.

At the last, the new type nerve conduit structure provides guiding cues and mechanical signals. In the context of PNI repair, particularly concerning extensive nerve defects, it is essential to facilitate the directed migration of stem cells and SCs, as well as to promote axonal regeneration. This aspect has emerged as a substantial area of interest in recent research on nerve conduits.

The influence of the physical environment on regenerative processes is important. Wang et al. [190] investigated the impact of nerve conduits with varying diameters (400 and 800 nm) and orientations (random versus aligned) on the treatment of PNI. Their findings indicated that the aligned nerve conduit with a diameter of 400 nm yielded the most favorable outcomes, enhancing the differentiation of human ESCs into neural progenitors and promoting the growth of neural axons. Similarly, Lewis et al. [191] examined nerve regeneration by incorporating ADSCs within a 3D hydrogel composed of uniaxially aligned collagen fibers. Their research revealed that aligned nerve conduits not only directed the organization of stem cells but also facilitated the deposition of ECM components and cytokines, thereby creating an improved microenvironment conducive to nerve regeneration. Furthermore, bioactive signals, including growth factors, surface modifications, and adhesion molecules, are critical in directing stem cell behavior and axonal regeneration [192]. Zhang et al. [193] developed a biodegradable chitosan/polycaprolactone (CS/PCL) nanofiber scaffold that incorporated melatonin and recombinant human NGF (rhNGF). As the conduit degrades, these bioactive agents are released, and both in vitro and in vivo studies demonstrated that the presence of rhNGF significantly enhances axonal regeneration and nerve repair. Based on the above, Yao et al. [194] integrated both physical and biological stimuli to develop a nerve conduit composed of reduced graphene oxide (rGO) and polycaprolactone (PCL), featuring a longitudinal topological structure infused with ADSCs. They harnessed the electroactive properties of rGO to activate the PI3K-Akt signaling pathway, thereby facilitating the differentiation of stem cells and mimicking the transmission of electrical signals in nerve tissue in vivo. Experimental results from animal models demonstrated that the performance of this conduit is on par with that of autologous nerve grafts. Future research endeavors should focus on investigating additional guiding cues and creating nerve conduits that incorporate a variety of stimuli, as this represents a great potential advancement in the field.

### Optimizing nerve conduit design for sustained release of exosomes

Exosome therapy is frequently constrained by rapid clearance and a limited half-life within the body [195], which hinders the sustained therapeutic effects of such treatments. Constructing a barrier to facilitate the gradual and continuous release of exosomes represents an innovative strategy. Researchers have utilized neural conduits infused with exosomes as a sustained release mechanism, but the distinctive architecture of the nerve conduit inhibits quick exosome discharge, thereby extending therapeutic benefits. Hydrogel materials appear to provide distinct benefits in this context.

Yang et al. [74] employed a conductive hydrogel infused with exosomes derived from BMSCs, which exhibit mechanical properties akin to those of natural neural tissue, alongside conductive capabilities. The engineered conductive hydrogel (ECH) interacts with the exosomes through hydrogen bonding, facilitating a sustained release mechanism for the exosomes. This ECH-Exo system not only fosters axonal regeneration but also mitigates inflammatory pain [74]. In a model of diabetic sciatic nerve compression injury, the treatment group receiving ECH-Exos demonstrated notable advantages in both histological and functional recovery [74].

Similarly, Liu et al. [195] utilized a decellularized pig sciatic nerve hydrogel (dDNH) that was loaded with exosomes (dExo) derived from differentiated hADMSCs exhibiting an SC phenotype. Their findings revealed that the dExo encapsulated within the dDNH exhibited a more sustained release profile compared to the direct application of dExo [195]. In the context of a sciatic nerve compression injury model, the dDNH loaded with dExo significantly enhanced recovery, as evidenced by improvements in both histological assessments and sensory and motor functions [195].

## Additional Physical Stimulation

To boost the efficacy of combination applications, researchers are not only focusing on exosomes, stem cells, and nerve conduits but also seeking to augment external stimulation to improve the adaptability and controllability of treatment approaches.

In the preliminary management of peripheral nerve restoration, noninvasive physical stimulation techniques (including electrical, magnetic, and laser treatments) showed advantageous outcomes. As surgical techniques emerged as the primary clinical therapy modality, researchers started investigations into the utilization of nerve conduits in conjunction with stem cell exosomes, examining the potential synergistic or augmentative effects of supplementary physical stimulation. Wu et al. [43] developed aligned nanofiber conduits composed of CNT/poly (p-dioxanone) (PPDO) and evaluated the differentiation and maturation efficacy of hADMSCs encapsulated within these conduits under conditions of CI, ES, and a combination of both. Their findings indicated that CI facilitates the secretion of stem cell-related growth factors by hADMSCs, while ES enhances the phenotypic maturation of hADMSCs into myelinated SC-like cells (SCLCs). The synergistic application of both methods further augmented phenotypic maturity, although the precise methodologies for their combination warrant further investigation. Concurrently, Du et al. [196] employed an in vitro ES testing apparatus to systematically examine the effects of polarity, electric potential, pulse frequency, and stimulation duration to identify the optimal ES protocol for promoting the differentiation of neural crest stem cells (NCSCs). Their research concluded that continuous stimulation at parameters of 200 mV/mm, 20 Hz, and 100 μs for a duration of 1 h constituted the most effective ES protocol. In subsequent animal studies, they observed that the nerve regeneration efficacy of NCSC transplantation, when paired with ES, was comparable to that achieved through autologous transplantation.

Xia et al. [6] have developed an innovative superparamagnetic multifunctional neural scaffold that integrates a magnetic gel containing superparamagnetic nanoparticles. This advancement facilitates the controlled in vivo release of EVs carried by stem cells through the modulation of a rotating magnetic field in vitro. The application of this magnetic gel for the regulation of exosome release could effectively address the challenge of low retention rates associated with exosomes. Additionally, Deng et al. [197] demonstrated that ultrasound can induce a fivefold increase in the release of exosomes from human astrocytes. If this property is also observed in cells commonly used for PNI, it may be possible to modulate exosome release by loading these cells onto hydrogel materials and applying ultrasound stimulation. In conclusion, the application of external physical stimuli to regulate exosome release from cells presents a promising strategy to mitigate the issue of low exosome retention rates.

## Discussion and Perspectives

Substantial efforts have been dedicated to enhancing the therapeutic outcomes associated with PNI. However, artificially synthesized nerve conduits have not demonstrated the same level of efficacy in clinical applications as autologous nerve grafts. Stem cells and exosomes, recognized as potent bioactive agents in tissue repair, have been substantiated by numerous studies for their roles in tissue regeneration mechanisms. Recently, there has been a growing body of research aimed at developing nerve conduits that integrate stem cells and exosomes to improve the clinical effectiveness of these conduits. This paper summarizes the advancements in the design of nerve conduits from 3 distinct perspectives: stem cells orexosomes, nerve conduits, and external physical stimulus. Furthermore, we explore prospective research avenues concerning the integration of stem cell-derived exosomes with nerve conduits, informed by the current state of research.

Stem cells have the capacity to enhance tissue repair through both paracrine signaling and differentiation, and they have been extensively utilized in the field of tissue engineering. To further advance the application of stem cells in conjunction with nerve conduits for the treatment of PNI, it is essential to consider both the source and functionality of the stem cells employed. Among the various sources, ADSCs are particularly advantageous due to their abundance; however, the techniques for their extraction require refinement. Traditional enzymatic digestion methods can compromise cell viability, whereas improved nonenzymatic approaches are still under development.

Exosomes are increasingly recognized as advantageous components in tissue engineering due to their inherent stability, biocompatibility, and low immunogenicity. As research in this field advances, it is imperative to develop methodologies for the large-scale production, separation, and characterization of exosomes to facilitate their integration into clinical applications. Furthermore, there is an urgent need to enhance the functionality of exosomes and to establish effective strategies for regulating their release. A great challenge in exosome research is the inadequate production levels [198]; thus, it is essential to optimize the culture medium composition, production systems, and production modalities for each specific cell line [198]. The integration of microfluidic technologies with immunoaffinity capture techniques holds promise for the efficient isolation of high-purity exosomes in future applications. Nevertheless, despite the availability of various isolation techniques, there remains a notable absence of standardized workflows from the isolation phase to characterization. As highlighted by Lai et al. [199] in accordance with the MISEV2018 guidelines, the establishment of standardized protocols for exosome isolation and characterization is crucial moving forward.

In the context of enhancing exosome functionality, the application of environmental stress to the source cells of exosomes produced unforeseen and excellent outcomes. Gene editing of cells derived from exosomes facilitated the overexpression of specific therapeutic agents, also resulting in satisfactory outcomes. Recent investigations into the use of exosomes as drug delivery vehicles have indicated a growing interest in RNA molecules, particularly miRNAs and siRNAs. However, further research is needed to elucidate the effects of miRNAs on the homeostasis of endogenous mRNAs and miRNAs, as well as to determine optimal dosages for miRNA-based therapies [200]. Besides genetic engineering, metabolic labeling serves as an excellent method for cellular functionalization [201]. Upon uptake by cells, metabolites are assimilated into the proteome, lipidome, DNA, and glycogen, indiscriminately altering biomolecules inside the cell. Consequently, exosomes are anticipated to transport metabolic markers. Alongside the use of cellular engineering techniques such as genetic engineering and metabolic labeling for exosome functionalization, direct modification of exosomes presents a viable approach. Three noncovalent strategies of multivalent electrostatic interactions, receptor–ligand binding, and hydrophobic insertion are extensively utilized to achieve stable changes of biological membranes [201]. Presently, prevalent bioconjugation reactions and “click chemistry” reactions that swiftly establish chemical bonds during the modification process have surfaced as potential methodologies for the covalent modification of exosomes. Researchers have successfully employed click chemistry to modify exosomes derived from M2 macrophages for spinal cord injury treatment, yielding promising results in animal models [202]. The application of click chemistry to modify the surface properties of exosomes may further enhance their efficacy in the repair of PNI.

Regarding the sustained or controlled release of exosomes, the development of hydrogel materials for nerve conduits has emerged as a critical advancement. Future efforts may involve the integration of various mechanical stimuli, such as electromagnetic forces, to optimize the controllability of exosome release in vitro. In summary, the advancement of exosome separation techniques and strategies has enabled the feasibility of exosome therapy, while functionalization and controlled release have enhanced its efficacy and manageability.

Furthermore, investigating the potential synergistic effects of various nerve conduit designs represents a critical advancement for future research. Currently, enhancements to nerve conduits, beyond the incorporation of stem cells and exosomes, encompass a range of studies that implement innovative strategies in drug delivery and controlled release, physical stimulation [203], catheter material properties [204], guiding cues [205], and other relevant factors. These results suggest that they enhance the healing efficacy of nerve conduits to some degree; nonetheless, there is a notable lack of research integrating these techniques with stem cell-derived exosomes. The future design of innovative nerve conduits with surface density gradient collagen particles, concurrently infused with stem cells and capable of remote exosome release using electromagnetic methods, is very promising. This method not only facilitates nerve regeneration but also more efficiently delivers biological signals to provide an improved regenerative microenvironment.

In conclusion, the integration of stem cells and exosomes with nerve conduits has the potential to remarkably improve the efficacy of nerve conduit repair, thereby facilitating their clinical utilization and serving as a primary focus in regenerative medicine research. Nevertheless, as previously indicated, there remains considerable opportunity for enhancement in these therapeutic approaches. Further comprehensive investigations are essential to develop and achieve treatment solutions for PNI that are more convenient, cost-effective, highly effective, and broadly applicable.

## Data Availability

Data sharing is not applicable to this article as no datasets were generated or analyzed during the current study.

## Supplementary Materials

Supplementary 1 Tables S1 and S2 References [206–221]
