# Supplementary material for: The Application of Stem Cells and Exosomes in Promoting Nerve Conduits for Peripheral Nerve Repair
Source: Biomater Res. 2025 Apr 14;29:0160. doi: 10.34133/bmr.0160 (PMC11994886; doi:10.34133/bmr.0160)
Supplement: Supplementary 1 — Tables S1 and S2 References [206–221] [file bmr.0160.f1.docx]

# The application of stem cells and exosomes in promoting nerve conduits for peripheral nerve repair

Mengen Li, ^1,2,3,4^# Ye Tang, 1,2,3,4# Chengkai Zhou,^1,2,3^ Yan Geng,^1,2,3^ Chenxi Zhang,^1,2,3^ YUWEI HSU,^1,2,3,5^ Le Ma,^1,2,3^ Wei Guo, ^5*^ Ming Li, 1,2,3* Yanhua Wang^1,2,4^*

1→National Center for Trauma Medicine, Beijing, 100044, China.

2→Key Laboratory of Trauma and Neural Regeneration, Ministry of Education, Peking University, Beijing, 100044, China.

3→Trauma Medicine Center, Peking University People's Hospital, Beijing, 100044, China.

4→Department of Orthopedics and Trauma, Peking University People's Hospital, Beijing100044, China.

5→Emergency Department, Peking University People's Hospital, Beijing, 100044, China.

*→Correspondence: woaiguowei111@sina.com (W.G.);liming_ort@bjmu.edu.cn (M.L.); 94719599@qq.com (Y.W.).

#→These author contributed equally to this work.

Supplementary materials

Table S1: The physiological functions and potential active components of exosomes in the treatment of peripheral nerve injury

| Origin of exosomes | Potential Active Components | Function | Reference |
| --- | --- | --- | --- |
| BMSCs | MiRNA-1260a, miRNA-21-5p, and miRNA-29b-3p | Enhancing vascular regeneration | ^[206-208]^ |
|  | MiRNA-23a, miRNA-125b | Alleviating neurovascular dysfunction | ^[209]^ |
| Differentiated human ADMSC with the Schwann cell phenotype | MiRNA-132-3p, miRNA-199b-5p | Protecting SCs from oxidative stress and enhanced HUVEC migration and angiogenesis | ^[92]^ |
| SCs | MiRNA-27a, miRNA-21, miRNA-164a | Promoting neurite outgrowth and migration of SCs | ^[210]^ |
| Endothelial cells | MiRNA199-5p | Maintaining repair-related phenotypes of SCs | ^[91]^ |
| Plasma | MiRNA-20b-3p | Alleviating autophagy impairment in SCs | ^[211]^ |

Table S2: The principles, advantages and disadvantages of methods based on microfluidic fields and immunocapture

| Isolation Technique | Mechanism | Advantage | Disadvantage | Reference |
| --- | --- | --- | --- | --- |
| Acoustic separation | Size | Fast, label-free, automatic, simple to operate, | Complex Specific instrumentation, difficulty in separating substances with similar acoustic characteristics to exosomes | ^[156,158,212]^ |
| Sieving separation | Size | Fast, label-free | Deformation and disruption, no guarantee for complete removal of contaminants | ^[156]^ |
| Electrical separation | Size | Label-free | Denaturation caused by excessive heat, less pure | ^[156,213]^ |
| Inertia separation | Size | Label-free | Hard to discriminate between exosomes and larger EVs | ^[156]^ |
| Viscoelastic separation | Size | Label-free, simple, high-throughput | Mixed with impurities, considered to be less helpful in separating | ^[156,214]^ |
| Deterministic lateral displacement sorting | Size, shape, deformability, charge | Label-free, low cost, simplicity, robustness, and precise manipulation of critical diameter | Time-consuming, low separation throughput, particles adhesion and clogging, complex and bulky experimental Setup | ^[156,215]^ |
| Electrical-Field-Flow Fractionation | Size, electrophoretic mobility | Label-free | Loss of exosome via adherence of the vesicles to the spin column and/or spin column filter | ^[156,216]^ |
| Centrifugal separation | Size, shape, density | Lable-free, gentle and faster than conventional centrifugation, label-free | Unable to separate continuously, need expensive platforms | ^[156]^ |
| ferrohydrodynamic separation | Size | Label-free, high purity | Low separation throughput，the impact of ferrofluids on the physiology of EVs remains unknown, unable to distinguish exosomes from impurities with similar sizes | ^[217]^ |
| Magneto-Immunoprecipitation | Size, specific binding | No upper limit of sample starting volume, simple to operate, no need for expensive equipment, high capture efficiency, high yield, high sensitivity | Difficulty in eluting intact from beads | ^[157,158,218,219]^ |
| Tim4@ILI-01 immunoaffinity material | Specific binding | high capture efficiency, high purify, with mild elution conditions |  | ^[161]^ |
| Asymmetric Flow Field-Flow Fractionation | Size | Label-free, simple, fast, preserve the integrity, high reproducibility, can distinguish subgroups of exosomes | Restricted sample loading volume, requirement for specialized equipment, ineffective in differentiating particles of similar size but varying morphology | ^[158,220,221]^ |
